# Reliability and Minimum Detectable Change of Temporal-Spatial, Kinematic, and Dynamic Stability Measures during Perturbed Gait

**DOI:** 10.1371/journal.pone.0142083

**Published:** 2015-11-04

**Authors:** Christopher A. Rábago, Jonathan B. Dingwell, Jason M. Wilken

**Affiliations:** 1 Center for the Intrepid, Department of Orthopaedics and Rehabilitation, Brooke Army Medical Center, JBSA Fort Sam Houston, Texas, United States of America; 2 Department of Defense and Veterans Affairs Extremity Trauma and Amputation Center of Excellence, JBSA Fort Sam Houston, Texas, United States of America; 3 Department of Kinesiology and Health Education, University of Texas at Austin, Austin, Texas, United States of America; Purdue University, UNITED STATES

## Abstract

Temporal-spatial, kinematic variability, and dynamic stability measures collected during perturbation-based assessment paradigms are often used to identify dysfunction associated with gait instability. However, it remains unclear which measures are most reliable for detecting and tracking responses to perturbations. This study systematically determined the between-session reliability and minimum detectable change values of temporal-spatial, kinematic variability, and dynamic stability measures during three types of perturbed gait. Twenty young healthy adults completed two identical testing sessions two weeks apart, comprised of an unperturbed and three perturbed (cognitive, physical, and visual) walking conditions in a virtual reality environment. Within each session, perturbation responses were compared to unperturbed walking using paired t-tests. Between-session reliability and minimum detectable change values were also calculated for each measure and condition. All temporal-spatial, kinematic variability and dynamic stability measures demonstrated fair to excellent between-session reliability. Minimal detectable change values, normalized to mean values ranged from 1–50%. Step width mean and variability measures demonstrated the greatest response to perturbations with excellent between-session reliability and low minimum detectable change values. Orbital stability measures demonstrated specificity to perturbation direction and sensitivity with excellent between-session reliability and low minimum detectable change values. We observed substantially greater between-session reliability and lower minimum detectable change values for local stability measures than previously described which may be the result of averaging across trials within a session and using velocity versus acceleration data for reconstruction of state spaces. Across all perturbation types, temporal-spatial, orbital and local measures were the most reliable measures with the lowest minimum detectable change values, supporting their use for tracking changes over multiple testing sessions. The between-session reliability and minimum detectable change values reported here provide an objective means for interpreting changes in temporal-spatial, kinematic variability, and dynamic stability measures during perturbed walking which may assist in identifying instability.

## Introduction

Perturbation-based, gait assessment paradigms can aid in identifying dysfunction often associated with age [1–3], disease [4, 5], or injury [6, 7]. Perturbations can be cognitive [1, 8–10], physical [6, 11–13], or visual [12, 14–16] in nature and are typically selected specific to deficits suspected in a population. In contrast, the temporal-spatial [3, 17–19], kinematic variability [13, 15, 20], and dynamic stability [12, 20, 21] measures collected during these assessments are often used interchangeably to quantify and characterize gait stability responses to perturbations. Unfortunately, the psychometric properties for many of these measures are not established for perturbation-based gait assessments, thus preventing their widespread utilization and the interpretation of published findings. Ideally, these measures would exhibit low minimal detectable change (MDC) values and strong between-session reliability to effectively identify deficits and track changes over time. Additionally, these measures should demonstrate sensitivity to expected gait responses across a range of commonly used perturbations.

During unperturbed gait, temporal-spatial measures like step width (SW), step length (SL), and stride time (ST) were reported to exhibit excellent between-session reliability (interclass correlation coefficient; ICC > 0.87) in healthy adults [22, 23]. Similarly, the between-session reliability of SW, SL, and ST was reported as excellent (ICC ≥ 0.75) when young adults walking at fast speeds (~1.60 m/s) were exposed to underfoot physical perturbations [24]. While between-session reliability was excellent, these results may not generalize to different types of perturbations. For example, between-session reliability for stride velocity variability decreased from moderate (ICC = 0.656) during unperturbed gait to poor (ICC = 0.226) during cognitively perturbed gait in older adults [25]. In contrast, ICC values for velocity and cadence measures remained excellent during both unperturbed and cognitively perturbed gait [25]. The authors suggested that measures of variability such as stride velocity variability might be inherently less reliable due to high between and within subject variance and may require hundreds of strides to increase between-session reliability during cognitively perturbed gait. To our knowledge, no group has reported the between-session reliability of temporal-spatial measures during visually perturbed gait.

As an alternative to temporal-spatial measures, multiple groups [26–32] have used local and orbital non-linear measures to assess dynamic gait stability, particularly in circumstances [12, 21, 33] and/or with populations [26, 27, 29, 32] where instability is prevalent. Local and orbital stability measures quantify how quickly responses to perturbations grow or decay over time. They have shown sensitivity and directional specificity in identifying gait responses to physical and visual perturbations [12, 33]. However, their utility in determining stability deficits in response to cognitive perturbations has proved inconclusive [20] and may require a large number of perturbed strides to detect deficits [34].

Recently, two groups have reported the within-session and between-session reliability of local dynamic stability measures (i.e. local divergence exponents) in healthy adults during unperturbed over ground [30, 35] and treadmill walking [36]. Short-term local dynamic stability measures were found to have good within-session reliability (ICC ≥ 0.70) and poor to fair between-session reliability (ICC ≤ 0.63) during over ground walking [35]. ICC and MDC values were strongly influenced by the state space reconstruction method employed. Similarly, short-term local dynamic stability measures were found to have greater within-session reliability (ICC ≥ 0.77) compared to between-session reliability (ICC ~ 0.60) during treadmill walking [36]. Further, short-term local dynamic stability measures demonstrated greater reliability than long-term local dynamic stability measures with both showing increased reliability as more strides were analyzed [36]. While these studies do provide some indication as to the reliability of local dynamic stability measures during unperturbed gait, to date, no study has reported both the between-session reliability and MDC of local and orbital stability measures during perturbed gait.

The general lack of reliability and MDC values for temporal-spatial, kinematic variability, and dynamic stability measures during perturbed gait limits their usability for quantifying, identifying, and tracking dysfunction. Further, the interpretation of published perturbation-based assessments findings is made more difficult without reliability and MDC reference values to help distinguish between differences associated with dysfunction and those due to measurement error. The purpose of this study was to systematically determine both the between-session reliability and MDC values of reported temporal-spatial, kinematic variability, and dynamic stability measures during perturbed and unperturbed gait. As part of this systematic investigation, three commonly used perturbations; color-interference Stroop (cognitive) [6, 19, 20, 34], walking surface oscillations (physical)[12, 15, 33, 37] and visual field oscillations (visual) [12, 15, 33, 37–41] were used to study the reliability and sensitivity of measured responses. Previous investigations indicate that the magnitude and type of responses may vary between selected measures and perturbation type [12, 15, 20], thus these factors are likely to affect reliability. The information from the present study could help facilitate the interpretation of other study results and improve the clinical utility of these measures during perturbation-based assessments.

## Methods

### Participants

Twenty participants (5 females, age: 26.1 ± 6.8 years, height: 1.75 ± 0.10 m, and mass: 78.3 ± 9.7 kg) completed two identical testing sessions. The experimental protocol was reviewed and approved by the Institutional Review Board at Brooke Army Medical Center. Written consent was obtained from each participant prior to enrollment. Exclusion criteria included neurologic and orthopedic injuries or disorders that would alter normal gait. Participants also had to pass a visual acuity and color discrimination screen. Glasses or contact lens were worn by participants who required them for corrected vision.

### Experimental apparatus

**Fig 1:**
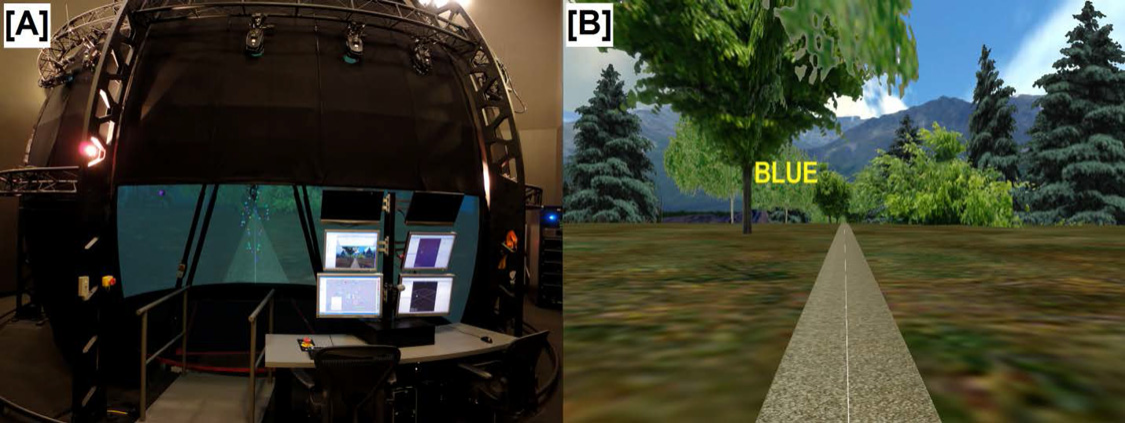
The Virtual Reality Environment. (A) Photograph of the virtual reality environment at the Military Performance Lab and (B) screen shot of Stroop walking scene where the word BLUE is displayed in yellow font.

All tasks were performed in a virtual reality environment (Computer Assisted Rehabilitation Environment; Motekforce Link, Amsterdam, Netherlands; Fig 1A) comprised of a 7 m diameter dome with 270-degrees of horizontal visual field projection and a 6-degrees of freedom motion platform [38]. Participants stood or walked in the center of a 1.8 x 2.8 m (width x length) instrumented treadmill wearing a safety harness tethered to a metal frame mounted outside their field of view. Full body 3-dimensional kinematic data were collected at 60 Hz using a 24-camera infrared motion capture system (Vicon Motion Systems, Oxford, UK) to track 57 reflective markers [33] place on hand, arm, head, trunk, pelvis, thigh, leg and foot segments.

### Experimental protocol

A wooded country scene with a centered walking path was displayed in the virtual reality environment during all conditions (Fig 1B). Participants were asked to walk down the path while maintaining a forward head orientation. In addition to unperturbed walking (NOP), participants walked while being perturbed (1) cognitively using a color-interference Stroop task (COG), (2) physically through translations of the walking surface (PLAT), or (3) visually with translations of the visual field (VIS). Three 3 minute trials of each gait condition were performed in random order and participants were allowed to rest in between trials. For all trials, each participant walked on the motorized treadmill in the virtual reality environment at the same constant speed scaled to their leg length:

$$speed=\sqrt{0.16\cdot g\cdot l}$$ (1)

where *g* = 9.81 m/s^2^ and *l* = leg length in meters [42]. Optic flow in the virtual reality environment was scaled to match the speed of the treadmill. Demonstrations, instructions, and practice time were provided before each condition to minimize learning effects. Participants returned for a second identical testing session within 14 ± 2 days.

The color-interference Stroop task consisted of words of common colors (i.e. red, blue, green, and yellow) displayed in an incongruent colored font (Fig 1B). Participants were required to avoid reading the word and instead report only the color of the font. Words were randomly displayed in front of the participant at a rate of one per second. The PLAT and VIS perturbation conditions consisted of continuous pseudo-random medial-lateral oscillations of either the visual scene or treadmill surface [12, 15]. Perturbations were applied as a pseudo-random sum of sines with 4 incommensurate frequencies (0.16, 0.21, 0.24 and 0.49Hz) in the following equation:

$$D\left(t\right)=A\left[\begin{array}{c} 1.0\;\text{sin}\left(0.16\cdot 2\pi t\right)+0.8\;\text{sin}\left(0.21\cdot 2\pi t\right)\\ +1.4\;\text{sin}\left(0.24\cdot 2\pi t\right)+0.5\;\text{sin}\left(0.49\cdot 2\pi t\right) \end{array}\right]$$ (2)

where D(t) is the translation distance (m), A is a scaling factor, and t is time (sec) [12, 15]. PLAT and VIS perturbations were scaled with A = 0.05 and A = 0.5 such that the maximum displacement was approximately 0.33m and 3.32m, respectively.

### Initial data processing

All data reduction and analyses were performed using Vicon Nexus 1.7 (Vicon Motion Systems, Oxford, United Kingdom), Visual3D (C-Motion Inc., Rockville, MD), and MatLab (The Mathworks, Natick, MA). Raw marker data were filtered with a zero-lag Butterworth filter at a low-pass cutoff frequency of 8 Hz. Heel strike and toe off events were determined using a velocity-based algorithm for comparing the anterior-posterior foot velocity relative to the pelvis [43].

### Temporal-spatial measures

Step length (SL) was defined as the distance between the foot centers in the anterior-posterior direction at heel strike. Step width (SW) was calculated as the medial-lateral, heel-to-heel distance between the two heel markers in double-limb stance. Stride time (ST) was quantified as the duration between consecutive heel strikes of the same foot. Means and standard deviations (SD) of SL, SW, and ST were calculated for each 3-minute walking trial.

### State Spaces

Delay embedded state spaces [44] were constructed using raw C7 vertebral marker velocity data and time delayed copies [45], such that:

$$S(t)=[v(t),v(t+\tau ),\ldots ,v(t+(d_{E}-1)\tau )]$$ (3)

where *S*(*t*) was the *d*_*E*_-dimensional state vector, *v*(*t*) was the original data, *τ* was the time delay and *d*_*E*_ was the embedding dimension. Time delays of 15, 10, and 30 samples, for anterior-posterior (AP), vertical (VT), and medial-lateral (ML) directions, respectively, were used as determined from assessments of the first minima of Average Mutual Information functions [46]. An embedding dimension of *d*_*E*_ = 5 [32] was used for all trials. To calculate orbital and local stability, state spaces were constructed using 124 continuous strides from each trial. For local stability analyses, these 124 continuous strides were first re-sampled to 12,400 total data points yielding an average of 100 data points per stride [34, 47] used during the delay embedding process.

### Orbital stability

Orbital stability was quantified by calculating the magnitude of the maximum Floquet multipliers (MaxFM), which quantify the rate of convergence or divergence from a limit cycle due to small perturbations, using established procedures [12, 48, 49]. If MaxFM > 1, a system is orbitally unstable as small perturbations would grow by the next cycle. Consequently, if MaxFM < 1, the system is orbitally stable indicating small perturbations diminish by the next cycle. Each delay embedded state space was divided into individual strides and each stride was time normalized to 101 samples, corresponding to 0–100% of the gait cycle. Poincare maps were defined at each percent of the stride as:

$$S_{k+1}=F(S_{\;k})$$ (4)

where *S* was the state of the system at stride *k* at each given Poincare section. The average trajectory for all strides in a given trial was used to define the fixed points as:

$$S^{*}=F(S^{*})$$ (5)

The orbital stability of the system was then quantified by estimating the Floquet multipliers by linearizing Eq 3 about these fixed points as:

$$\left[S_{k+1}-S^{*}]≅J(S^{*})[S_{k}-S^{*}\right]$$ (6)

where *J*(S*) is the Jacobian matrix for the system at each Poincare section. The eigenvalues of each of the 101 *J*(S*) are the Floquet multipliers. Orbital stability was then defined as the maximum value from the magnitudes of the largest eigenvalues (i.e., MaxFM).

### Local stability

Local stability was quantified by calculating the local divergence exponents. Nearest neighbor points in the reconstructed state space represent the effects of small local perturbations to the system. The local divergence exponents quantify the response of a system to small local perturbations [12, 20, 50, 51]. The local divergence exponents (LDE) were estimated using the slopes of linear fits to the mean log divergence curve:

$$y(i)=\frac{1}{\Delta t}\langle \text{ln}[d_{j}(i)]\rangle =[\lambda^{*}]i+c$$ (7)

where *d*_*j*_(*i*) was the Euclidean distance between the *j* ^*th*^ pair of initially nearest neighbors after *i* discrete time steps (i.e., *i*Δ*t* seconds) and 〈∙〉 denotes the average over all values of *j*. Short-term (λ*_S_) and long-term (λ*_L_) LDE were calculated as the slopes of the linear fits of the divergence curve between 0 and 1 stride and between 4 and 10 strides [52], respectively. Positive LDE indicate local instability.

### Trunk kinematic variability

Trunk kinematic variability during walking conditions was characterized using C7 marker velocities in the AP, VT, and ML directions [15, 20]. Data for each individual stride were time normalized to 101 samples, corresponding to 0–100% of the gait cycle. Standard deviations were calculated across all strides at each time normalized point within a single trial. Standard deviations were then averaged over the normalized stride to yield the *MeanSD* for each trial using:

$$MeanSD(V_{x})=\langle SD_{n}[V_{x}]\rangle$$ (8)

where *V*_*x*_ is the velocity in each direction (i.e., *x* ∈{*AP*, *ML*, *VT*}), *n* indicates each time normalized point of the gait cycle (0%, …, 100%), and 〈∙〉 indicates the average over all *n* [20, 45].

### Statistical analyses

Temporal-spatial, kinematic variability, and dynamic stability means and standard deviations were used to describe group response magnitudes during the unperturbed and perturbed conditions. For each measure, within session differences between the unperturbed and each of the three perturbed conditions were evaluated using three paired t-tests. A Bonferroni-Holm correction was performed to correct for these multiple comparisons. The Bonferroni–Holm method uses a step-down approach to account for multiple comparisons by arranging p-values from the smallest to the largest and comparing them to sequential significance cutoffs [53]. A correction factor accounting for the three comparisons was applied with the smallest p-value cutoff set to 0.05/3 = 0.0167.

Between-session differences (i.e. session 1 vs. session 2) in temporal-spatial, kinematic variability, and dynamic stability measures were determined using paired t-tests. Effect size was determined for each comparison using the Cohen d statistic using the following equation:

$$d=t_{s}\cdot \sqrt{2\cdot \left(1-r\right)/N}$$ (9)

where *t*_*s*_ is the effect size t-value from the comparisons, r is the correlation between the comparisons, and N is the number of participants [54]. This equation uses the correlation coefficient to limit overestimation of the effect magnitude. An effect size of 0.2–0.3 signifies a “small” effect, 0.5–0.7 a “medium” effect, and ≥ 0.8 a “large” effect [55].

The between-session (i.e. session 1 vs. session 2) reliability (ICC) of each temporal-spatial, kinematic variability, and dynamic stability measure was calculated using a two-way random model (2, k) for consistency [56]. ICC values ≥ 0.75 were considered “excellent”, 0.40–0.74 “fair to good”, and < 0.40 “poor” [57]. In order to calculate MDC values, the standard error of the measurement (SEM) was first determined using the equation

$$SEM=SD\times \sqrt{\left(1-ICC\right)}$$ (10)

where *SD* is the standard deviation from the first testing session. *MDC* values were calculated using the equation:

$$MDC=SEM\times 1.96\times \sqrt{2}$$ (11)

[58]. SEM and MDC values were determined for each temporal-spatial, kinematic variability, and dynamic stability measure using Microsoft Excel 2007 (Microsoft Corp., Redmond, WA). All t-tests and *ICC* calculations were performed using SPSS Statistics 19.0 (IBM, www.spss.com).

## Results

### Temporal-spatial measures

**Fig 2:**
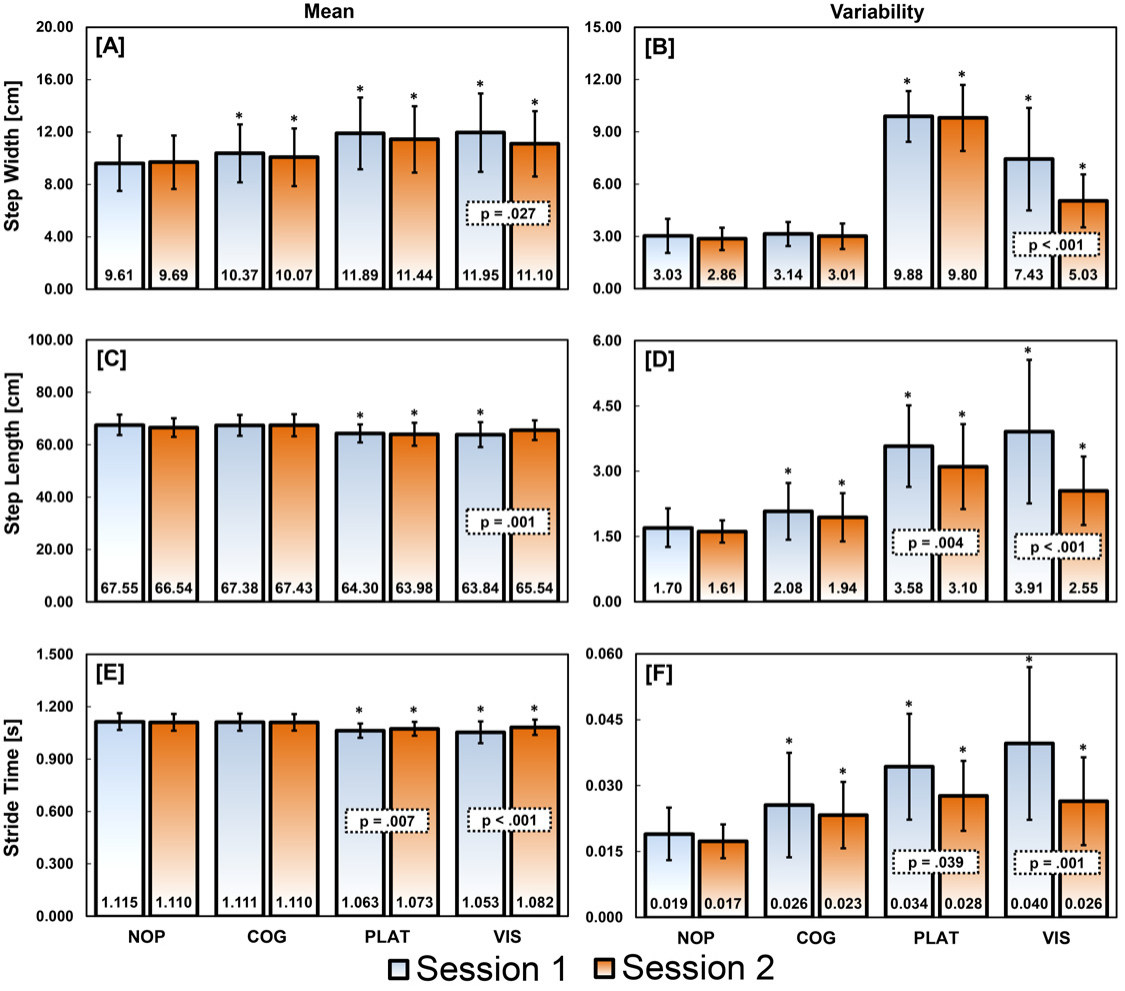
Temporal-spatial means and variability values during unperturbed and perturbed walking. Walking conditions are labeled as NOP–no perturbation, COG–cognitive perturbation, PLAT–physical perturbation, and VIS–visual perturbation. Group means and standard deviations are depicted for each measure, condition, and session. Significant differences (p < 0.05) between the unperturbed and each perturbed walking condition during the same session are labelled with an *. Significant differences between sessions for each measure of the same condition are highlighted with a dotted box containing calculated p values.

Participants walked at an average speed of 1.20 ± 0.04 m/s across all walking conditions. In response to all perturbation conditions, participants tended to walk with increased SW, SW variability, SL variability, and ST variability and decreased SL and ST (Fig 2 and Table 1). Reponses exhibited during COG were smaller than PLAT and VIS. Significant differences (p < 0.05) were seen during COG in SW mean, SL variability, and ST variability compared to NOP. During both sessions, the effect sizes for SW mean and SL variability were small to medium, while the ST variability differences exhibited large effect sizes (Table 1). PLAT and VIS conditions elicited the largest responses with mean and variability values for SW, SL, and ST demonstrating significant differences (p < 0.05) compared to NOP. In general, the effect sizes for these differences were large during both sessions (Table 1).

**Table 1:** Temporal-spatial p-values and effect sizes (Cohen’s d) for paired unperturbed to perturbed condition comparisons and paired between-session comparisons.

p-value [Cohen’s d]	NOP1-COG1	NOP2-COG2	NOP1-PLAT1	NOP2-PLAT2	NOP1-VIS1	NOP2-VIS2	NOP1-NOP2	COG1-COG2	PLAT1-PLAT2	VIS1-VIS2
Step Width Mean (cm)	**0.001 [0.35]**	**0.027 [0.17]**	**0.001 [0.85]**	**0.001 [0.72]**	**0.001 [0.82]**	**0.001 [0.56]**	0.634 [0.04]	0.164 [0.13]	0.082 [0.18]	**0.027 [0.30]**
Step Width Variability (cm)	0.466 [0.12]	0.079 [0.21]	**0.001 [5.09]**	**0.001 [3.72]**	**0.001 [1.89]**	**0.001 [1.65]**	0.270 [0.19]	0.302 [0.18]	0.651 [0.04]	**0.001 [0.78]**
Step Length Mean (cm)	0.673 [0.04]	0.269 [0.20]	**0.001 [0.86]**	**0.003 [0.67]**	**0.001 [0.82]**	0.049 [0.31]	0.160 [0.23]	0.761 [0.03]	0.585 [0.12]	**0.001 [0.45]**
Step Length Variability (cm)	**0.049 [0.63]**	**0.048 [0.48]**	**0.001 [2.26]**	**0.001 [1.97]**	**0.001 [1.77]**	**0.001 [1.65]**	0.163 [0.32]	0.096 [0.40]	**0.004 [0.46]**	**0.001 [0.92]**
Stride Time Mean (s)	0.708 [0.04]	0.918 [0.01]	**0.001 [1.14]**	**0.001 [0.84]**	**0.00 [1.14]**	**0.001 [0.55]**	0.772 [0.03]	0.913 [0.01]	**0.007 [0.38]**	**0.001 [0.68]**
Stride Time Variability (s)	**0.006 [1.03]**	**0.001 [1.04]**	**0.001 [1.27]**	**0.001 [1.37]**	**0.001 [1.49]**	**0.001 [1.12]**	0.419 [0.24]	0.359 [0.18]	**0.039 [0.60]**	**0.001 [0.88]**

Walking conditions are labeled as NOP–no perturbation, COG–cognitive perturbation, PLAT–physical perturbation, and VIS–visual perturbation. The numbers 1 and 2 indicate conditions performed during session one and two, respectively. Bold values depict significant differences.

Significant between sessions differences (p < 0.05) were observed for the VIS condition with a change in all temporal-spatial measures toward NOP values (Fig 2 and Table 1). The effect sizes for these differences ranged from small (d = 0.30) to large (d = 0.92, Table 1). These between-session differences had a negative effect on reliability with VIS demonstrating lower between-session reliability for SW, SW variability, and ST variability compared to the other conditions (Table 2). Overall, temporal-spatial measures during PLAT demonstrated the best reliability with 5 of the 6 measures exhibiting ICC values in the excellent range (0.83–0.95). Further, SW, SW variability, SL, and ST measures demonstrated excellent reliability across all unperturbed and perturbed conditions.

**Table 2:** Temporal-spatial ICC and MDC values during unperturbed and perturbed walking.

ICC [MDC]	NOP	COG	PLAT	VIS
Step Width Mean (cm)	**0.96** [0.58]	**0.96** [0.63]	**0.95** [0.81]	**0.91** [1.22]
Step Width Variability (cm)	**0.80** [0.58]	**0.83** [0.39]	**0.94** [0.50]	**0.79** [1.80]
Step Length Mean (cm)	**0.87** [1.90]	**0.97** [0.93]	**0.83** [1.88]	**0.94** [1.54]
Step Length Variability (cm)	0.49 [0.43]	0.54 [0.59]	**0.89** [0.42]	0.61 [1.39]
Stride Time Mean (s)	**0.98** [0.010]	**0.96** [0.013]	**0.94** [0.031]	**0.91** [0.026]
Stride Time Variability (s)	0.34 [0.007]	0.65 [0.009]	0.38 [0.013]	0.58 [0.015]

Walking conditions are labeled as NOP–no perturbation, COG–cognitive perturbation, PLAT–physical perturbation, and VIS–visual perturbation. Bold values exhibited excellent between-session reliability (ICC ≥ 0.75).

**Fig 3:**
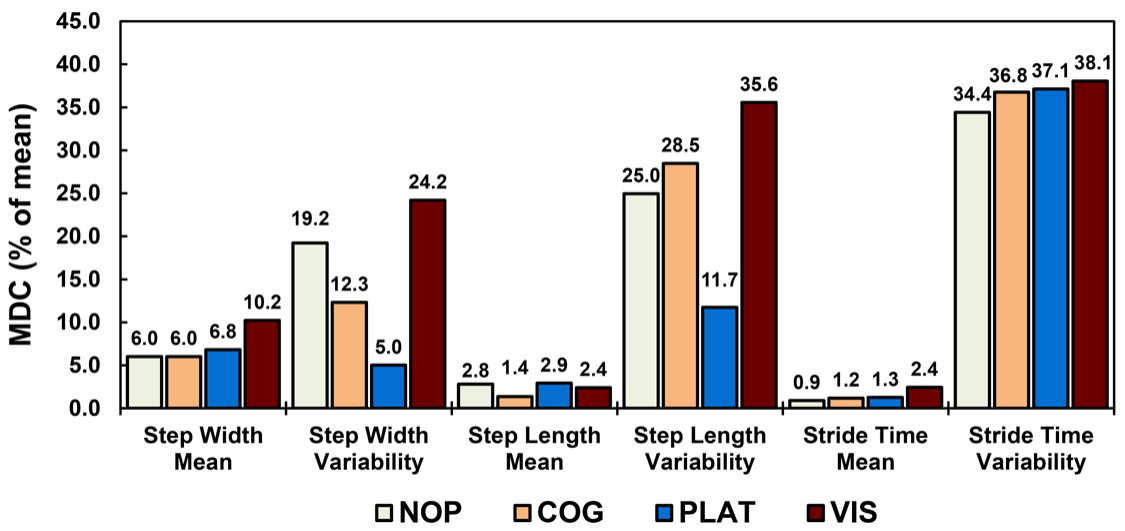
Temporal-spatial MDC values during unperturbed and perturbed walking. Walking conditions are labeled as NOP–no perturbation, COG–cognitive perturbation, PLAT–physical perturbation, and VIS–visual perturbation. Values shown as percent of measurement mean.

The significant differences identified between the unperturbed and perturbed conditions were all above the MDCs calculated for each temporal-spatial measure (Table 2). In general, mean temporal-spatial gait measures exhibited lower MDC values compared to variability measures when normalized to group means (Fig 3). Furthermore, all temporal-spatial gait measures for the PLAT condition consistently demonstrated MDC (percent of mean) values comparable to or less than the NOP condition (Fig 3).

### Trunk variability and stability measures

**Fig 4:**
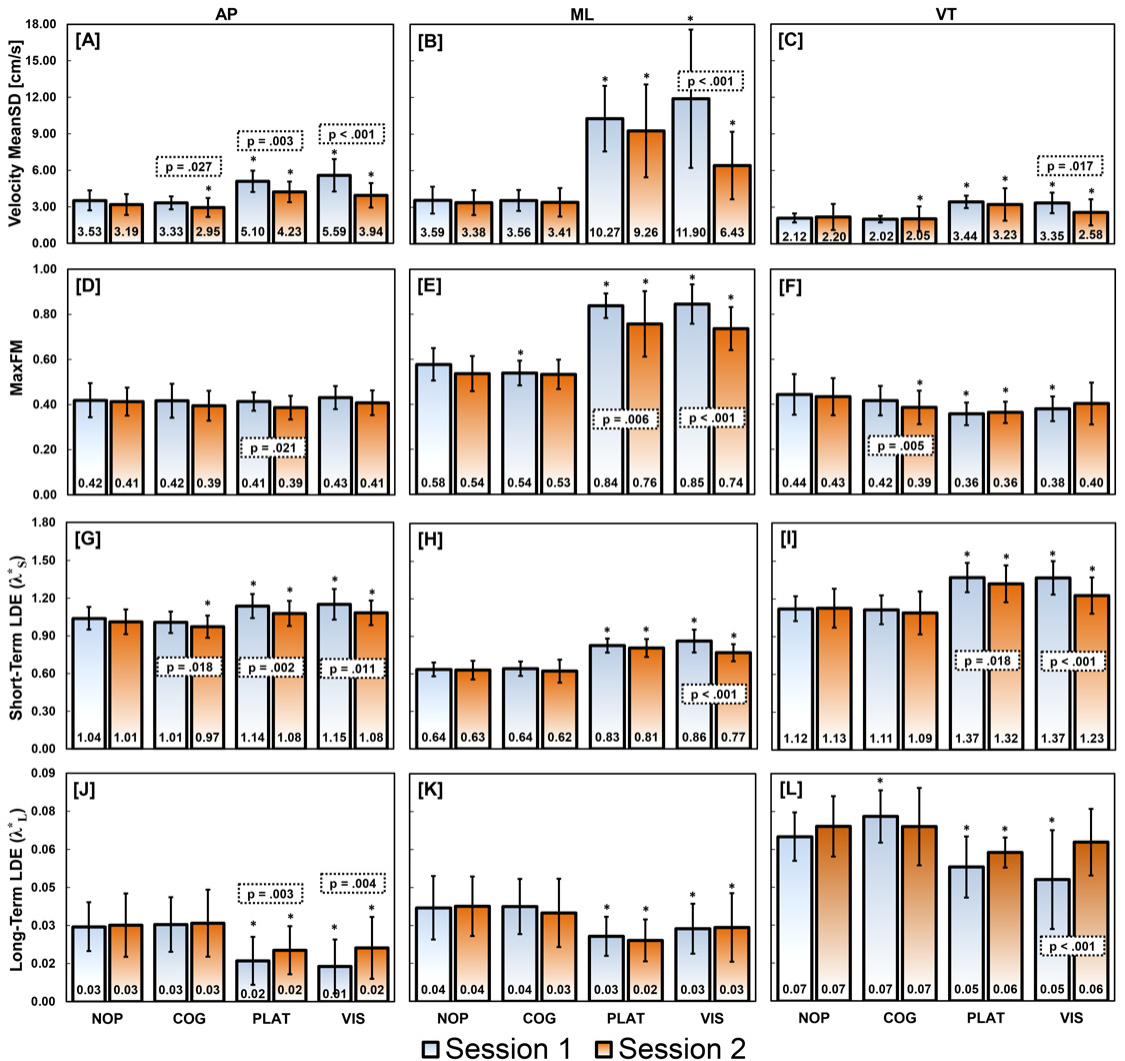
Trunk kinematic variability and stability measures during unperturbed and perturbed walking. Walking conditions are labeled as NOP–no perturbation, COG–cognitive perturbation, PLAT–physical perturbation, and VIS–visual perturbation. Group means and standard deviations are depicted for each measure, condition, session, and direction of motion: anterior-posterior (AP), medial-lateral (ML), and vertical (VT). Significant differences (p < 0.05) between the unperturbed and each perturbed walking condition during the same session are labelled with an *. Significant differences between sessions for each measure and condition are highlighted with a dotted box containing calculated p values.

In general, participants exhibited similar trunk kinematic variability (i.e. velocity MeanSD) and dynamic stability (i.e. MaxFM and LDE) during the COG and NOP conditions (Fig 4 and Table 3). In contrast, trunk movement variability and instability significantly increased (p < 0.003) with PLAT and VIS compared to NOP with medium to large effects sizes (Fig 4 and Table 3). These differences were greatest in the ML direction compared to the AP. Further, significant increases in MaxFM occurred primarily in the oscillation direction (i.e. ML) during PLAT and VIS conditions. In contrast, significant differences in LDE were seen in all 3 principle directions during PLAT and VIS conditions.

**Table 3:** Trunk movement variability and dynamic stability p-values and effect sizes (Cohen’s d) for paired unperturbed to perturbed condition within-session comparisons and paired between-session comparisons.

p-value [Cohen’s d]	NOP1-COG1	NOP2-COG2	NOP1-PLAT1	NOP2-PLAT2	NOP1-VIS1	NOP2-VIS2	NOP1-NOP2	COG1-COG2	PLAT1-PLAT2	VIS1-VIS2
AP										
Velocity MeanSD (cm/s)	0.281 [0.29]	**0.028 [0.29]**	**0.001 [1.84]**	**0.001 [1.22]**	**0.001 [1.76]**	**0.001 [0.79]**	0.237 [0.41]	**0.027 [0.56]**	**0.003 [1.01]**	**0.001 [1.40]**
MaxFM	0.909 [0.03]	0.342 [0.28]	0.700 [0.09]	0.091 [0.46]	0.466 [0.17]	0.668 [0.09]	0.659 [0.09]	0.062 [0.31]	**0.021 [0.57]**	0.101 [0.44]
Short-term LDE (λ*_S_)	0.076 [0.37]	**0.003 [0.41]**	**0.001 [1.04]**	**0.001 [0.67]**	**0.001 [0.98]**	**0.001 [0.73]**	0.193 [0.30]	**0.018 [0.40]**	**0.002 [0.60]**	**0.011 [0.60]**
Long-term LDE (λ*_L_)	0.714 [0.08]	0.780 [0.06]	**0.001 [1.41]**	**0.001 [0.86]**	**0.001 [1.54]**	**0.001 [0.73]**	0.741 [0.05]	0.809 [0.04]	**0.003 [0.44]**	**0.004 [0.63]**
ML										
Velocity MeanSD (cm/s)	0.881 [0.03]	0.818 [0.02]	**0.001 [3.00]**	**0.001 [1.58]**	**0.001 [1.93]**	**0.001 [1.93]**	0.545 [0.19]	0.575 [0.14]	0.055 [0.27]	**0.001 [1.02]**
MaxFM	**0.002 [0.58]**	0.835 [0.05]	**0.001 [4.09]**	**0.001 [1.70]**	**0.001 [3.34]**	**0.001 [2.27]**	0.096 [0.55]	0.746 [0.10]	**0.006 [0.56]**	**0.001 [1.19]**
Short-term LDE (λ*_S_)	0.583 [0.09]	0.431 [0.09]	**0.001 [3.41]**	**0.001 [2.40]**	**0.001 [2.96]**	**0.001 [1.94]**	0.717 [0.09]	0.310 [0.24]	0.164 [0.29]	**0.001 [1.12]**
Long-term LDE (λ*_L_)	0.860 [0.04]	0.444 [0.21]	**0.001 [1.03]**	**0.001 [1.30]**	**0.014 [0.73]**	**0.003 [0.65]**	0.792 [0.05]	0.180 [0.20]	0.407 [0.20]	0.875 [0.04]
VT										
Velocity MeanSD (cm/s)	0.206 [0.30]	**0.001 [0.14]**	**0.001 [2.91]**	**0.001 [0.72]**	**0.001 [1.85]**	**0.001 [0.35]**	0.754 [0.10]	0.899 [0.04]	0.501 [0.21]	**0.017 [0.80]**
MaxFM	0.134 [0.34]	**0.011 [0.61]**	**0.001 [1.06]**	**0.001 [1.00]**	**0.001 [0.69]**	0.065 [0.34]	0.575 [0.12]	**0.005 [0.42]**	0.507 [0.13]	0.163 [0.28]
Short-term LDE (λ*_S_)	0.577 [0.08]	0.090 [0.23]	**0.001 [2.27]**	**0.001 [1.29]**	**0.001 [2.08]**	**0.005 [0.67]**	0.840 [0.03]	0.421 [0.16]	**0.018 [0.35]**	**0.001 [1.01]**
Long-term LDE (λ*_L_)	**0.010 [0.80]**	0.984 [0.01]	**0.003 [1.10]**	**0.002 [1.08]**	**0.002 [1.09]**	0.089 [0.50]	0.229 [0.37]	0.271 [0.30]	0.078 [0.60]	**0.001 [0.86]**

Walking conditions are labeled as NOP–no perturbation, COG–cognitive perturbation, PLAT–physical perturbation, and VIS–visual perturbation. The numbers 1 and 2 indicate conditions performed during session one and two, respectively. Values shown in each direction of motion: anterior-posterior (AP), medial-lateral (ML), and vertical (VT). Bold values depict significant differences.

Significant between sessions differences (p < 0.05) were observed for the PLAT and VIS conditions with a change in most variability and stability measures toward NOP values (Fig 4 and Table 3). This change was greatest in the ML direction during Visual perturbations with large effect sizes (d > 1.02, Table 3). However, the between-session reliability (Table 4) for trunk velocity MeanSD, MaxFM, and short-term LDE in the ML direction were greater during VIS (ICC > 0.70) compared to the NOP condition (ICC < 0.59). Overall, between-session reliability for all trunk variability and stability measures improved during the perturbed conditions compared to the NOP.

**Table 4:** Trunk movement variability and dynamic stability ICC and MDC values during unperturbed and perturbed walking.

ICC [MDC]	NOP			COG			PLAT			VIS		
Direction	AP	ML	VT	AP	ML	VT	AP	ML	VT	AP	ML	VT
Velocity MeanSD (cm/s)	-0.31 [1.27]	0.04 [1.45]	0.03 [0.49]	0.60 [0.45]	0.51 [0.80]	-0.09 [0.38]	0.25 [1.02]	**0.87** [1.28]	0.09 [0.65]	-0.08 [1.86]	0.70 [4.19]	0.13 [1.06]
MaxFM	0.74 [0.051]	0.03 [0.095]	0.73 [0.063]	**0.86** [0.038]	0.19 [0.067]	**0.90** [0.028]	0.65 [0.033]	0.61 [0.045]	**0.76** [0.033]	0.53 [0.047]	0.71 [0.063]	0.70 [0.040]
Short-term LDE (λ*_S_)	0.66 [0.070]	0.59 [0.048]	**0.84** [0.053]	**0.86** [0.043]	0.60 [0.049]	0.73 [0.080]	**0.83** [0.053]	0.72 [0.040]	**0.88** [0.054]	0.70 [0.090]	0.70 [0.067]	0.68 [0.101]
Long-term LDE (λ*_L_)	**0.86** [0.005]	**0.80** [0.008]	0.18 [0.012]	**0.81** [0.006]	**0.88** [0.005]	0.42 [0.011]	**0.91** [0.004]	0.60 [0.007]	-0.09 [0.017]	**0.77** [0.007]	0.49 [0.009]	0.60 [0.017]

Walking conditions are labeled as NOP–no perturbation, COG–cognitive perturbation, PLAT–physical perturbation, and VIS–visual perturbation. Values shown in each direction of motion: anterior-posterior (AP), medial-lateral (ML), and vertical (VT). Bold values exhibited excellent between-session reliability (ICC ≥ 0.75).

**Fig 5:**
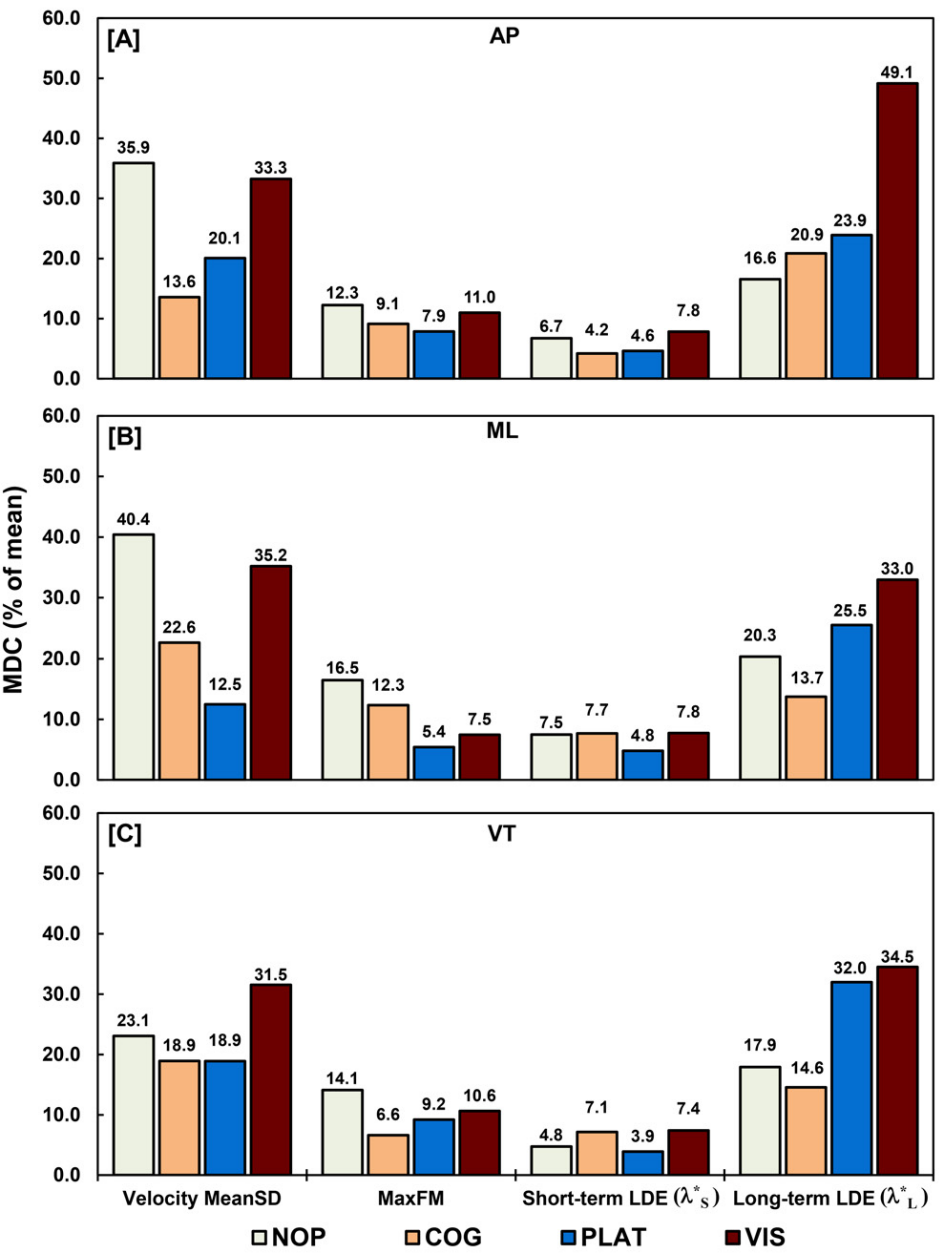
MDC for trunk kinematic variability and stability measures during unperturbed and perturbed walking. Walking conditions are labeled as NOP–no perturbation, COG–cognitive perturbation, PLAT–physical perturbation, and VIS–visual perturbation. Values shown as percent of measurement mean for each direction of motion: anterior-posterior (AP), medial-lateral (ML), and vertical (VT).

The significant differences identified between the unperturbed and perturbed conditions were all above the MDCs calculated for each variability and stability measure (Table 4). In general, MaxFM and short-term LDE measure exhibited lower MDC values compared velocity MeanSD and long-term LDE measures when normalized to group means (Fig 5). Trunk velocity MeanSD, MaxFM and short-term LDE measures for the PLAT condition consistently demonstrated MDC (percent of mean) values comparable to or less than the NOP condition (Fig 5).

## Discussion

Gait performance during perturbations can be used to assess an individual’s ability to effectively respond to challenges. Gait responses identified will often depend on population specific deficits, but can also be strongly affected by the psychometric properties of the gait measure selected. However, it remains unclear which measures most consistently detect corrective responses. Therefore, we systematically determined the between-session reliability and MDC values of commonly reported temporal-spatial, kinematic variability, and dynamic stability measures during three different types of perturbed walking. Interclass correlation coefficient (ICC) and minimum detectable change (MDC) values in healthy young adults were calculated to provide an objective assessment of reliability and facilitate the selection of gait measures during perturbation-based assessments.

### Temporal-spatial measures

The temporal-spatial differences observed between perturbation and NOP conditions were all above calculated MDCs (Fig 2 and Table 2) signifying true gait changes in response to perturbations that were not due to chance. In general, these differences indicated that participants walked with shorter, wider, and quicker steps that were also more variable when exposed to perturbations (Fig 2). These findings closely match previous reports which used similar visual and physical perturbations in virtual reality environments [11, 15]. In contrast, observed responses to COG did not match the results of Grabiner and Troy [19] who used an identical Stroop task to perturb gait. Specifically, they reported a non-significant 4% decrease (p = 0.10) in mean SW and a significant 16% decrease (p = 0.029) in SW variability while performing the Stroop task during gait. Our participants during session 1 exhibited a significant 8% increase (p = 0.001, d = 0.35) in mean SW compared to NOP with no significant change in SW variability. The addition of optic flow to our Stoop task may have assisted participants in maintaining their gait heading [59], thus mitigating responses to the Stroop. Also, we postulate that the larger area of our treadmill allowed participants to adopt a larger step width without needing to make corrective steps at the sides of the treadmill. On a narrower treadmill belt with rails (as described in [10]), participants might potentially need to more tightly control step width and possibly make more corrections to avoid walking off the sides [19] or touching the hand rails. This illustrates how the physical environment, regardless of the perturbation properties, can influence gait responses and possibly the reliability of their measurement.

The type of perturbation modality used can also affect gait response over time and may change the between-session reliability of temporal-spatial measures. For example, participants walking in a virtual reality environment were reported [37] to adapt to visual field oscillations in as little as one exposure. In our study, temporal-spatial measures from the second VIS session normalized toward values observed in the NOP condition suggesting a habituation to a repeated exposure of visual field oscillations. The between-session differences observed during the VIS condition contributed to small reductions in ICC and %MDC values. However, all temporal-spatial measures remained significantly different from NOP values indicating continued gait alteration in response to the visual perturbations that were above our calculated MDCs. Despite an expected habituation to visual perturbations, the temporal-spatial measures demonstrated excellent between-session reliability (ICC ≥ 0.75, Table 2) with MDC values sufficient for tracking treatment effects to visual perturbations. Besides their use during assessments, visual perturbations have been used to address balance impairments associated with visuo-spatial deficits in conditions like traumatic brain injury [60–62]. Our reported MDCs are important as they give clinical researchers a means to objectively interpret differences seen between treatment and assessment sessions.

Perturbation-based assessment paradigms and temporal-spatial measures are often used to identify deficits during ambulation often associated with instability [3, 17–19]. However, the relationship between gait instability and changes in temporal-spatial mean and variability measures is still unclear. Temporal-spatial mean differences may indicate the presence of compensatory strategies in response to perturbations while changes in variability may represent positive adaptations to destabilizing conditions. Of the temporal-spatial measures reported here, step width mean [3] and variability [63] are said to be greater discriminators of instability compared to step length and stride time measures. Older adults who fell, and thus consider unstable, were found to have increased mean step widths compared to younger adults suggesting a compensatory strategy to increase balance by widening base of support [64]. Further, older adults who fell displayed decreased step width variability compared to older adults with no history of falls (i.e. increased stability) [64] suggesting a diminished ability to vary responses to destabilizing conditions. In this study, none of our young participants display instability responses which may have led to a fall suggesting that they were capable of producing appropriate compensations and adaptations. Mean measures did exhibited greater ICC and lower %MDC values compared to variability measures across all perturbation types (Fig 3) which may indicate consistency in the compensatory strategy employed during the perturbations. Specifically, participants continued to walk with shorter, wider, and quicker steps during the second session but variability decreased suggesting that participants did not need vary compensatory strategies as much during the second session. While this may point to learning or adaptation to the perturbation over time, it is important to note that the significant difference between perturbed and NOP conditions above MDC values were still observed in the second session.

The temporal-spatial measures studied here demonstrated excellent between-session reliability (ICC ≥ 0.75) with the exception of SL and ST variability. In the second session compared to the first, SL and ST variability decreased across all conditions resulting in slightly lower ICC values compared to the other measures. The reduction in SL and ST variability may indicate that subjects were able to improve gait speed control to better match treadmill speed. When walking on a treadmill, gait control is thought to be organized around a goal function that produces a gait velocity equal to the fixed treadmill speed [65, 66]. A “Goal Equivalent Manifold” (GEM) can be defined as all combinations of SL and ST which yield the treadmill speed. Humans have been shown to minimize errors relative to this GEM by making small consistent stride-to-stride changes in SL and ST during non-perturbed [66] gait in order to remain on the treadmill’s surface. In contrast, when young adults walking on a treadmill were challenged with cognitive perturbations, variability in the non-goal-equivalent direction of the GEM increased [65]. This meant that more combinations of SL and ST that produced velocities not equivalent to the treadmill speed were observed during perturbations. Similarly, we measured a significant increase in SL and ST variability during perturbations compared to the non-perturbed condition signifying gait velocity inconsistencies. During the perturbation conditions, participants were observed to drift backwards on the treadmill belt suggesting a preference to slow their walking velocity. However, they had to speed up once they drifted too far back in order to stay on the treadmill belt. The ability to drift on the treadmill was likely due to its large surface area that allowed for gait speeds over several strides that were slower than the treadmill. The decrease in SL and ST variability during the second session most likely reflected improved gait speed control even during perturbed gait. This improvement may also reflect a learning effect following repeated exposures which enforced speed control in order to maintain a positioning on the treadmill.

### Trunk variability and dynamic stability measures

Compared to the temporal-spatial measures, between-session reliability for trunk kinematic variability and dynamic stability measures varied over a greater range of ICC values and were, generally, not as high. Of these measures, short-term (λ*_S_) and long-term (λ*_L_) LDE are the only measures to have reliability and MDC values reported in the literature [30, 35, 36]. Between-session ICC for λ*_S_ were poor to moderate (≤ 0.63) and MDCs ranged from 17% to 46% of their mean with both strongly influenced by the state space reconstruction method utilized [35]. The best between-session reliability and MDC values achieved were with state space reconstructions using fixed time delays (6, 24 samples), embedding dimensions (7, 9), and 200 strides [35]. We used a similar state space reconstruction method, albeit with slightly different time delays (15, 10, 30 samples), embedding dimensions (5), and number of strides (124 per trial). With our reconstruction method, we observed between-session ICC values for λ*_S_ that were fair to excellent (≥ 0.59) with MDCs ranging from 3.9% to 7.8% (Fig 5) across all non-perturbed and perturbed conditions. In addition, we observed between-session ICC values for λ*_L_ that were fair to excellent (0.49–0.91) with %MDCs ranging from 16.6% to 49.1% in ML and AP directions across all conditions. In contrast, a previous report found between-session reliability (ICC: 0.47–0.67) and %MDCs (67–107%) for λ*_L_ to be considerably poorer than λ*_S_ [36]. We report substantially greater between-session reliability and lower %MDCs for λ*_S_ and λ*_L_ than previously described which may be the result of using velocity versus acceleration [30, 35, 36] data for reconstruction of the state spaces. The characteristics (e.g. variability and noise) are likely different across these kinematic signals which may have improved λ*_S_ and λ*_L_ values reliability [35]. Between-session reliability and MDCs may have been further improved because we chose to average λ*_S_ and λ*_L_ values across the three trials in each session. Reported ICC values indicate good (≥ 0.67, [30, 35]) to excellent (≥ 0.84, [36]) λ*_S_ within-session reliability which improved as more strides were analyzed [36] supporting the averaging of λ*_S_ and λ*_L_ during a single session to better estimate local dynamic stability.

Trunk kinematic variability (i.e. velocity MeanSD) and orbital stability (i.e. MaxFM) between-session reliability and MDC values have not previously been reported in the literature. Compared to λ*_S_ and λ*_L_, velocity MeanSD and MaxFM between-session reliability and MDCs values were less consistent across non-perturbed and perturbed conditions. In general, velocity MeanSD and MaxFM reliability and MDCs values improved during PLAT and VIS conditions where responses demonstrated directional specificity to walking surface and visual field ML perturbations. In contrast, local stability (i.e. LDE) measures exhibited less directional specificity with similar responses in all directions. These results are in agreement with reports of Floquet multipliers demonstrating greater specificity in their responses to perturbation direction [12] compared to LDE. Floquet multipliers were also stated to have less sensitivity during walking surface and visual field perturbations [12] compared to LDE. However, if %MDC is considered a measure of sensitivity to change, MaxFM demonstrated %MDCs of 5.4% and 7.5% in the ML direction during PLAT and VIS (Fig 5). These values are near equivalent to the %MDCs of λ*_S_ (4.8%, 7.8%) and much smaller than λ*_L_ (25.5%, 33.0%) in the ML direction during PLAT and VIS. Thus, MaxFM demonstrated specificity to perturbation direction and sensitivity that can be used to detect and track changes in response to physical and visual perturbations. Between-session reliability for velocity MeanSD was the highest (ICC = 0.87, 0.70) in the ML direction during PLAT and VIS conditions. However, MDCs were only low during the PLAT condition. Similar to the temporal-spatial measures during the VIS condition, velocity MeanSD MDCs increased possibly due to habituation to the visual oscillations over the two sessions.

Similar to other reports [20, 34], trunk kinematic variability and dynamic stability measures demonstrated little sensitivity to cognitive perturbations. We found during COG of the first session that MaxFM in the ML direction was significantly different than NOP. Also, during the second session COG condition, we observed significant decreases in velocity MeanSD and λ*_S_ values. These differences were below our calculated MDCs for each measure suggesting that these differences maybe be the result of biological variation and measurement error. In a similar study using the Stroop as a cognitive perturbation, changes in dynamic stability could only be detected using a substantial number of strides (>150) [34].

While trunk kinematic variability and dynamic stability measures during the COG condition compared to the NOP did not appear to differ much, between-session reliability and MDC values did improve. The presence of the Stoop task may have offered subjects a more specific point to focus on visually that facilitated their ability to maintain gait heading. This may help explain the higher level of consistency across the two sessions [59]. Further, young healthy participants have also been shown to prioritize gait at the expense of cognitive performance [9, 10, 19, 67] when presented with a cognitive challenge during gait. Thus, the low level challenge of the Stroop task may have contributed to the small effects observed [20] and the improved between-session reliability and MDC values. For future studies, cognitive tasks with internal interfering factors like mental tracking or arithmetic problems may elicit greater effects on gait than those with external interfering factors such as the Stroop task [68].

## Conclusions

In this present study, temporal-spatial, MaxFM, and LDE measures were the most reliable measures with the lowest MDC values across all perturbation types, supporting their use for tracking changes over multiple testing sessions. Of the temporal-spatial measures, SW mean and variability measures demonstrated the greatest response to perturbations with excellent between-session reliability and low MDCs. MaxFM demonstrated specificity to perturbation direction and sensitivity with excellent between-session reliability and low MDC values supporting their use in detecting and tracking changes in response to physical and visual perturbations. We report substantially greater between-session reliability and lower %MDCs for λ*_S_ and λ*_L_ than previously described which may be the result of using velocity versus acceleration [30, 35, 36] data for reconstruction of the state spaces and averaging across trials within a session.

The three perturbation conditions described in this study were chosen because of their frequent use with temporal-spatial, trunk kinematic, and dynamic stability measures in identifying deficits during perturbation-based gait assessments [11, 12, 15, 18, 19, 34]. They represent three types of perturbations (i.e. cognitive, physical, and visual) often described during dual-task studies [59, 68–72], but do not encompass all perturbations reported in the literature. Cognitive, physical, and visual perturbation-based assessments each provide unique information for evaluating gait performance, as response magnitude and measure reliability vary by perturbation type.

In this study, we established between-session reliability and MDC values not previously reported for temporal-spatial, trunk kinematic, and dynamic stability measures during perturbed gait. These measures demonstrated fair-excellent reliability across three types of perturbations. Often, between-session reliability and MDC values are specific to the application, presentation environment, instructions given, and subject populations as they are affected by biological variability and methodological error. While investigators will likely want to determine between-session reliability and MDC values for each unique application and population, the values reported here provide normative (i.e. young healthy) reference data to assist in the interpreting of changes observed during perturbed walking in populations (i.e. elderly, non-healthy) with histories of gait instability. Further study would be necessary to determine what specific effects that factors like learning, adaptation, perturbation type/direction, and state space reconstruction methods may have on the reliability of the temporal-spatial, kinematic variability, and dynamic stability measures.
